# Soil Metagenome Sequences from a Geothermal Site at Mount Melbourne in Antarctica

**DOI:** 10.1128/MRA.01490-20

**Published:** 2021-03-11

**Authors:** Mira Park, Yong-Hoe Choe, Mincheol Kim

**Affiliations:** a Institute of Basic Sciences, Incheon National University, Incheon, Republic of Korea; b Korea Polar Research Institute, Incheon, Republic of Korea; Indiana University, Bloomington

## Abstract

Here, we examined two soil metagenome data sets obtained from a geothermal site at the summit of Mount Melbourne in Antarctica. The geothermal soil microbiome exhibits very unique features of prokaryotic diversity and functions, which will provide deeper insight into the adaptation and evolution of soil microbes in Antarctic geothermal habitats.

## ANNOUNCEMENT

Mount Melbourne (2,733 m above sea level) is a quiescent stratovolcano located in North Victoria Land facing the Ross Sea in Antarctica. Ice-free areas occur on the upper parts of the volcano due to geothermal activity, and cryptogams are commonly found around the ridges or fumarolic steam vents. Most biological studies on these geothermal habitats have been directed toward the macroscopic flora, such as bryophytes, lichens, and algae (1). Prokaryotic diversity in this area remains poorly understood because most studies have been conducted using traditional culture-dependent approaches (2, 3). Over the past decade, molecular characterization of microbial communities inhabiting fumarolic soils on Mount Erebus demonstrated that prokaryotic diversity in Antarctic geothermal habitats is much greater than previously thought (4, 5), which increases the possibility of detecting more cryptic lineages endemic to Mount Melbourne through molecular methods.

Two surface mineral soil samples (0- to 5-cm depth) spaced approximately 3 m from each other were collected from a geothermal site at the summit of Mount Melbourne (74°21.334′S, 164°41.733′E) on 25 November 2018 using a sterile trowel. Soil DNA was extracted from 0.3 g of each soil sample using a DNeasy PowerSoil Pro kit (Qiagen, Germany). Sequencing libraries were prepared using the TruSeq Nano DNA library preparation kit (Illumina, USA), and paired-end reads (31.6 Gbp for site 1 and 32.8 Gbp for site 2) were obtained from shotgun metagenomic sequencing using a HiSeq 4000 system (2 × 150 bp) at Macrogen, Inc. (Seoul, South Korea). Sequencing adapters and low-quality reads were filtered out by Trimmomatic v0.39 with default settings (6). Quality-trimmed sequences were searched against the NCBI nonredundant database (downloaded on 29 September 2020) using the DIAMOND BLASTX aligner v0.9.10.111 (options: -b12 -c1 -e 0.00001) (7), and then the search results were mapped to Kyoto Encyclopedia of Genes and Genomes (KEGG) (July 2020) and Genome Taxonomy Database (GTDB) (release 95) mapping files in MEGAN6 Ultimate Edition v6.19.8 for taxonomic and functional classification (8). Taxonomic analysis revealed that most sequences were represented by bacteria (97.0%), and archaea accounted for only a minor fraction (0.2%) of the total prokaryotic community. The dominant bacterial phyla were *Dormibacterota* (candidate phylum AD3) (23.1% on average), *Chloroflexota* (20.8%), *Proteobacteria* (17.3%), *Acidobacteriota* (12.7%), *Planctomycetota* (5.7%), and *Actinobacteriota* (5.2%). Less abundant phyla included *Gemmatimonadota* (4.8%), candidate phylum CSP1-3 (4.2%), *Methylomirabilota* (1.6%), and *Cyanobacteria* (1.3%), and rare phyla (representing <0.5% of the sequences) included *Bacteroidota* (0.4%), *Verrucomicrobiota* (0.4%), *Firmicutes* (0.4%), *Nitrospirota* (0.3%), and *Thermoproteota* (0.3%). Functional analysis revealed that genes involved in CO_2_ fixation through the Calvin-Benson-Bassham and reductive tricarboxylic acid cycles were similarly abundant. Genes encoding all of the Sox enzymes (SoxXYZABCD) were detected, suggesting the potential for complete thiosulfate oxidation to sulfate by the Sox pathway.

### Data availability.

Raw metagenome sequence data were deposited in the NCBI Sequence Read Archive (SRA) under the accession numbers SRR13258664 and SRR13254584 (BioProject number PRJNA685225).

## References

[1] Broady P, Given D, Greenfield L, Thompson K. 1987. The biota and environment of fumaroles on Mt. Melbourne, Northern Victoria Land. Polar Biol 7:97–113. doi:10.1007/BF00570447.

[2] Bargagli R, Skotnicki ML, Marri L, Pepi M, Mackenzie A, Agnorelli C. 2004. New record of moss and thermophilic bacteria species and physico-chemical properties of geothermal soils on the northwest slope of Mt. Melbourne (Antarctica). Polar Biol 27:423–431. doi:10.1007/s00300-004-0612-6.

[3] Nicolaus B, Marsiglia F, Esposito E, Trincone A, Lama L, Sharp R, Di Prisco G, Gambacorta A. 1991. Isolation of five strains of thermophilic eubacteria in Antarctica. Polar Biol 11:425–429. doi:10.1007/BF00233077.

[4] Soo RM, Wood SA, Grzymski JJ, McDonald IR, Cary SC. 2009. Microbial biodiversity of thermophilic communities in hot mineral soils of Tramway Ridge, Mount Erebus, Antarctica. Environ Microbiol 11:715–728. doi:10.1111/j.1462-2920.2009.01859.x. 19278453

[5] Herbold CW, Lee CK, McDonald IR, Cary SC. 2014. Evidence of global-scale aeolian dispersal and endemism in isolated geothermal microbial communities of Antarctica. Nat Commun 5:3875. doi:10.1038/ncomms4875. 24846491

[6] Bolger AM, Lohse M, Usadel B. 2014. Trimmomatic: a flexible trimmer for Illumina sequence data. Bioinformatics 30:2114–2120. doi:10.1093/bioinformatics/btu170. 24695404 PMC4103590

[7] Buchfink B, Xie C, Huson DH. 2015. Fast and sensitive protein alignment using DIAMOND. Nat Methods 12:59–60. doi:10.1038/nmeth.3176. 25402007

[8] Huson DH, Auch AF, Qi J, Schuster SC. 2007. MEGAN analysis of metagenomic data. Genome Res 17:377–386. doi:10.1101/gr.5969107. 17255551 PMC1800929

